# Acquired Isolated Factor VII Deficiency in Plasma Cell Dyscrasias: A Brief Presentation of Two Plasma-Cell-Leukemia-Related Cases and Review of Literature

**DOI:** 10.3390/jcm12185837

**Published:** 2023-09-08

**Authors:** Anna Furlan, Francesca Sartori, Filippo Gherlinzoni

**Affiliations:** Hematology Unit, Azienda ULSS2 Marca Trevigiana, 31100 Treviso, Italy; francesca.sartori@aulss2.veneto.it (F.S.); filippo.gherlinzoni@aulss2.veneto.it (F.G.)

**Keywords:** factor VII deficiency, plasma cell disorders, multiple myeloma, AL amyloidosis, acquired coagulopathy, acquired bleeding disorders, PT prolongation, recombinant activated factor VII

## Abstract

Acquired isolated factor VII (FVII) deficiency is a rare but important discovery in patients with plasma cell disorders with significant therapeutic and prognostic implications. The present analysis and review of cases reported in the literature is intended to highlight disease-related characteristics associated with this rare clotting defect, clinical manifestations and outcome, and potential underlying mechanisms, and to provide guidance on how to manage these patients in terms of prophylactic and therapeutic measures. The discovery of acquired FVII deficiency in a patient with multiple myeloma (MM) or monoclonal gammopathy of uncertain significance (MGUS) should prompt an evaluation for AL amyloidosis, particularly for amyloid hepatosplenic involvement, whenever not previously documented. Acquired FVII deficiency in patients with MM and AL amyloidosis is frequently associated with severe bleeding diathesis, also related to a number of concomitant predisposing factors, adversely affecting the outcome. The prompt institution of a rapidly acting therapy is crucial to prevent severe bleeding complications and positively impact outcome. Recombinant activated factor VII (rVIIa) may represent a useful supportive care measure, both in treating active bleeding and in the peri-procedural setting. However, further clinical experience is needed to optimize the therapeutic management of this rare disorder.

## 1. Case Presentation

### 1.1. Case 1

A 52-year-old woman was diagnosed with multiple myeloma (MM), IgA/k, ISS stage III, in April 2023. Her past medical history was significant for arterial hypertension and depressive syndrome, both of which were treated pharmacologically.

The bone marrow biopsy revealed a 70% monotypic plasma cell infiltration expressing CD138+, CD38+, and B-cell differentiation markers (CD20+, PAX5+). Fluorescent in situ hybridization (FISH) turned out to be positive for t(11;14) in 95% of nuclei and negative for high risk cytogenetic abnormalities [del17p, gain1q, t(4;14), t(14;16), and t(14;20)]. Molecular analysis was positive for TP53 mutation (exon 5).

At the beginning of induction therapy with D-VTd (Daratumumab, Bortezomib, Thalidomide, and Dexamethasone), blood counts showed ≥5% of circulating plasma cells in peripheral blood, within the context of rapidly progressing pancytopenia, were consistent with primary plasma-cell leukemia (pPCL). A low dose of aspirin (100 mg/dL) for prophylaxis of venous thomboembolism was given, as per the guidelines [1].

The day after starting therapy, the patient was admitted to hospital for massive epistaxis. Aspirin was discontinued, as well as Thalidomide, due to the contraindication to antithrombotic prophylaxis.

Laboratory examinations on admission showed Hb 6.4 g/dL, WBC 2.04/mm^3^ with ANC 1080/mm^3^, circulating plasma cells 6%, platelets 46.000/mm^3^, creatinine 0.92 mg/dL, calcium 8.7 mg/dL, AST 24 U/L, ALT 12 U/L, total bilirubin 0.6 mg/dL (direct 0.4 mg/dL), γGT 43 U/L, ALP 174 U/L (nv 35–105), LDH 76 U/L, total serum protein 11.4 g/dL, serum M-protein 3.6 g/dL, IgA/k, IgG 397 mg/dL, IgA 7196 mg/dL, IgM 26 mg/dL, serum free light chains (sFLC) k/λ 930.65/6.53, ratio 142.159, NT-proBNP 8522 pg/mL (nv 0–125 pg/mL), PT ratio 1.5, PTT 34.9 s, AT 65%, and fibrinogen 119 mg/dL. No data regarding the coagulation profile prior to hospital admission were available.

A total body CT scan revealed multiple osteolytic lesions and hepatosplenomegaly (maximum bipolar spleen diameter 15.5 cm; maximum bipolar liver right lobe diameter 25 cm); no significant mediastinal or intra-abdominal lymphadenopathies were documented.

An attempt to cauterize an actively bleeding varix in the left nostril was unsuccessful, and nasal tamponade was required repeatedly over the following 5 days.

In an attempt to correct the coagulopathy, fresh frozen plasma (FFP) replacement (15–20 mL/kg) was given daily, along with platelet transfusions (2 to 3 units per week to target a threshold of 30.000/mm^3^) and tranexamic acid (1000 mg IV TID), without succeeding in controlling bleeding. Transfusion of packed red cells was required daily over the first week in hospital due to severe anemia. Despite massive FFP infusion, PT ratio remained prolonged (1.5–1.7) in the face of complete correction of fibrinogen levels. A 3-day course of IV vitamin K also failed to correct the PT. A PT mixing test indicated a corrected result, suggesting a factor deficiency as the cause of the PT prolongation in the absence of an inhibitor. FVII level turned out to be low at 22%. Factor II, V, VIII, X, and von Willebrandt Ag levels were all within normal limits.

The hospital stay was initially complicated by congestive heart failure, which was resolved after diuretic therapy with furosemide.

An early treatment response assessment at the completion of cycle 1 D-VTd showed serum M-protein at 5.3 g/dL, consistent with progressive disease. The patient then developed severe acute respiratory distress secondary to bilateral interstitial pneumonia associated with Cytomegalovirus infection, requiring transfer to the Intensive Care Unit and invasive ventilation. Over the next few days she developed progressive liver dysfunction, likely secondary to rapidly progressive disease with liver involvement (AST 61 U/L, ALT 53 U/L, total bilirubin 8.7 mg/dL, direct 8.1 mg/dL, albumin 2.3 mg/dL). In the face of worsening liver dysfunction, PT ratio remained stable and PTT and fibrinogen were within normal limits throughout the course of hospitalization. The patient died four weeks after hospital admission due to a massive intracranial hemorrhage that was not susceptible to surgical treatment.

Hepatosplenomegaly, ALP, and NT-proBNP elevation at diagnosis were highly suggestive of hepatosplenic and cardiac AL amyloidosis. Red Congo staining performed on the bone marrow biopsy specimens proved negative. Abdominal fat and hepatic biopsy to definitely rule out amyloidosis were not performed due to the high risk of bleeding and to the critical clinical conditions, especially considering the fact that the detection of amyloidosis was unlikely to impact the therapeutic approach.

### 1.2. Case 2

A 54 year-old man was diagnosed with MM, IgG/λ, in 2019. He was treated with VTd (Bortezomib, Thalidomide, and Dexamethasone) induction therapy followed by autologous stem cell transplantation (SCT) consolidation and Lenalidomide maintenance. Subsequent lines of therapy at first and second relapse were IsaKd (Isatuximab, Carfilzomib, and Dexamethasone) and EloPd (Elotuzumab, Pomalidomide, and Dexamethasone), respectively. FISH analysis performed at second relapse was positive for del17p and gain1q, both in 80% of nuclei.

After 2 EloPd cycles, he was admitted to hospital due to fever, abdominal pain, and pancytopenia with severe anemia. An esophagogastroduodenoscopy showed esophageal candidiasis and ruled out active bleeding. Laboratory examinations on admission were consistent with disease progression evolving to secondary plasma-cell leukemia (sPCL): Hb 7.2 g/dL, WBC 1.2/mm^3^ with ANC 490/mm^3^, circulating plasma cells 5%, platelets 23.000/mm^3^, creatinine 0.75 mg/dL, calcium 7.6 mg/dL, AST 25 U/L, ALT 21 U/L, total bilirubin 1.4 mg/dL (direct 0.8 mg/dL), γGT 22 U/L, ALP 228 U/L (nv 35–105), LDH 370 U/L (nv 135–214), total serum protein 8.7 g/dL, serum M-protein 3.8 g/dL, IgG/λ, IgG 4146 mg/dL, IgA 12 mg/dL, IgM 8 mg/dL, sFLC k/λ 8.02/13828, ratio 0.001, serum albumin 1.9 mg/dL, total urine protein 910 mg/24 h, Bence-Jones positive, λ, NT-proBNP12285 pg/mL (nv 0–125), Troponin T 51.0 ng/L (nv 0–14), PT ratio 1.85, PTT 29.4 s, AT 77%, fibrinogen 589 mg/dL, and CRP 26.7 mg/dL. Red Congo staining performed on the abdominal fat biopsy proved negative.

**Figure 1:**
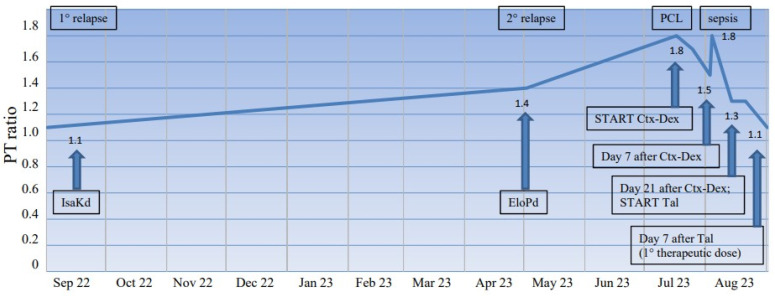
Time course of PT ratio in patient 2 throughout the disease history. PT ratio was normal at the time of MM diagnosis (not shown) and at first relapse. A modest increase in PT ratio (1.4) emerged at second relapse, progressively rising to 1.8 over the next two months concurrently with the evidence of progression to PCL. Partial correction of PT was documented 7 days after the start of Ctx-Dex. Worsened PT prolongation and decreased FVII level (22%) were associated with sepsis during chemotherapy-induced neutropenia. Three weeks after Ctx-Dex were administered, the patient was started on Tal. Complete correction of PT ratio (1.1) was documented one week after the first therapeutic dose of Tal. Abbreviations: Ctx-Dex = Cyclophosphamide and Dexamethasone; EloPd = Elotuzumab, Pomalidomide, and Dexamethasone; IsaKd = Isatuximab, Carfilzomib, and Dexamethasone; PCL = plasma-cell leukemia; Tal = Talquetamab.

Notably, PT ratio was normal at the time of MM diagnosis and at first relapse. A modest increase in PT ratio (1.4) emerged at second relapse, progressively rising to 1.8 over the next two months concurrently with the evidence of progression to plasma-cell leukemia (Figure 1). 

The isolated PT prolongation was evaluated by means of a PT mixing test indicating a corrected result, consistent with the absence of an inhibitor. FVII level proved to be low at 44%, while factor II, V, and X levels were all within normal limits. No clinical signs of cardiac dysfunction or liver congestion were present.

An abdominal ultrasound showed the liver to be within normal limits and revealed an enlarged spleen (maximum bipolar diameter 15 cm), both with homogeneous ecostructure.

Despite the coagulopathy and concomitant thrombocytopenia, the patient manifested a mild bleeding tendency, mostly in the form of easy bruising. An attempt to correct PT with FFP (20 mL/kg) in preparation for a follow-up esophagogastroduodenoscopy failed, as did a 2-day course of IV vitamin K. Prophylaxis with IV tranexamic acid was given during the stay in the hospital.

Pending the availability of bispecific antibody Talquetamab, in view of the rapid disease progression and the related bleeding risk, Cyclophosphamide (1500 mg IV on days 1 and 3) and a high dose of Dexamethasone (40 mg IV on days 1–4) were administered as a bridge to immunotherapy, with partial correction of PT documented 7 days after the start of treatment. Worsened PT prolongation and decreased FVII level (22%), without PTT or fibrinogen abnormalities, were associated with Carbapenemase-producing *Klebsiella pneumoniae* bloodstream infection during chemotherapy-induced neutropenia. Three weeks after Cyclophosphamide and Dexamethasone were administered, the patient was started on Talquetamab. Complete correction of PT ratio (1.1) was documented one week after the first therapeutic dose of Talquetamab. The time course of PT ratio throughout the disease history is reported in Figure 1.

To the best of our knowledge, these are the first reports of acquired isolated FVII deficiency in two patients with primary and secondary PCL, respectively. Although the diagnosis of amylodosis remains unconfirmed and an evidence-based explanation for the coagulopathy could not be given, these case presentations are intended to highlight the association of prolonged PT secondary to acquired isolated FVII deficiency and hepatosplenomegaly in the context of a biologically aggressive plasma cell disorder such as plasma-cell leukemia, and the relation between clinical course of coagulopathy and response to the underlying disease treatment.

## 2. Introduction: Acquired Isolated FVII Deficiency, a Rare but Potentially Significant Finding

Acquired bleeding conditions are often the result of the deficiency of several clotting factors. Acquired isolated defects of clotting factors are rare conditions. The most common and widely known is factor X (FX) deficiency, associated with AL amyloidosis. Systemic light chain (AL) amyloidosis is a clonal plasma cell disorder characterized by the extracellular deposition of fibrils composed of monoclonal immunoglobulin light chains. It presents alone or in association with other plasma cell and related disorders such as MM or Waldenström macroglobulinemia. AL amyloidosis is associated with multi-system clinical and laboratory manifestations resulting from amyloid deposition in various organs and tissues, such as the heart, kidneys, liver, spleen, gastrointestinal tract, skin, and nerves.

Amyloidosis-associated FX deficiency has been hypothesized to be secondary to the rapid clearance of factor X from the circulating blood through the absorption and immobilization of the protein to amyloid fibrils in the vasculature, mainly in the liver and spleen [2]. Studies [3,4,5,6] have reported that 8.7 to 14% of patients with AL amyloidosis had factor X levels of less than 50% of the normal levels, and 56% of those with reduced levels had clinically significant bleeding episodes [3]. However, FX deficiency does not account entirely for coagulopathy and bleeding diathesis in AL amyloidosis. A larger proportion of amyloidosis patients, approximately one third, exhibit bleeding symptoms, and about half, overall, show abnormal clotting findings [4]. Coagulation abnormalities have been reported to be associated with advanced disease [5,6], liver involvement, and inferior outcomes [6]. FX deficiency, in particular, has been proven to correlate with higher disease stage, the involvement of more than one organ, liver, and cardiac involvement, and greater than 10% bone marrow plasma cells [6].

Acquired isolated FVII deficiency is rare, in contrast to congenital FVII deficiency, which is the most frequent disorder among the rare congenital coagulation defects [7]. FVII is a coagulation factor dependent on vitamin K and is synthesized in the liver. It is usually found in human plasma at concentrations of 0.5 mcg/mL, with a relatively short half-life of approximately 3–4 h in the circulation, although this may be shorter during a bleeding episode [8]. FVII is often found in the shape of an inactive single-chain zymogen, and it is present in the circulation at the rate of 1% in an activated form (FVIIa). Vascular injury results in the binding of FVII to tissue factor (TF). FVII bound to TF is activated to generate the active serine protease FVIIa and it is the TF–VIIa complex that, through limited proteolytic cleavage, activates factors X and IX and the subsequent coagulation cascade [7,9].

In a review of literature including 29 patients with acquired isolated FVII deficiency published in 2016 by Girolami et al., underlying conditions were reported to be cancer, infections, polytrauma, penicillin administration, nephritic syndrome, Wiskott Aldrich syndrome, and left heart failure [10]. Among the 14 patients with neoplastic disease, 6 were diagnosed with hematological malignancies, including 1 case of systemic amyloidosis. Notably, of the six reported cases associated with hematologic malignancies (myelofibrosis, acute myeloid leukemia (AML), acute lymphoblastic leukemia (ALL), and chronic myeloid leukemia (CML)), four occurred in the setting of myeloablative chemotherapy and allogenic stem cell transplantation (SCT). Toor et al. reported a retrospective series of eight patients who presented with PT prolongation secondary to acquired FVII deficiency in the first 2 weeks following autologous or allogenic SCT. The incidence of bleeding complications and the rate of mortality directly due to hemorrhage is high in this particular setting [11].

## 3. Characteristics of Patients with Acquired Isolated FVII Deficiency Associated with Plasma Cell Disorders

In a retrospective study of 411 patients with AL amyloidosis, FVII deficiency was reported in 57% of patients, in the face of a prevalence of PT abnormalities of 19%. Notably, abnormality was defined as less than 65% for FVII (median: 46%). Therefore, FVII deficiency defined as less than this threshold, and did not invariably translate into PT prolongation. Moreover, the prevalence and impact of FVII as an isolated abnormality were not reported in this study [6].

Seven cases of acquired isolated FVII deficiency associated with plasma cell disorders (MM/PCL and/or AL amyloidosis) are reported in the literature [12,13,14,15,16], including the two personal cases here described, and one patient with combined FVII and FX deficiency [17]. The analysis also includes a patient taken from our case records with AL amyloidosis and isolated PT prolongation, whose abnormality resolved immediately after splenectomy for splenic rupture, but who was not specifically tested for FVII activity. None of these patients had a history of clotting abnormalities and or bleeding diathesis before the diagnosis of plasma cell disorder. Patient characteristics, clinical course, treatments, and outcomes are summarized in Table 1.

Five out of nine cases are associated with AL amyloidosis (+/− MM). Interestingly, all amyloidosis patients presented with liver and/or spleen involvement. Overall, in six out of nine cases, including the three personal cases reported here, hepatosplenomegaly has been documented, in two cases evolving to splenic rupture. Accordingly, the analysis by Abdallah et al. revealed that, compared with AL amyloidosis patients with normal PT, patients with a prolonged PT were more likely to have liver involvement (33% vs. 15%) [6]. In four cases of MM/PCL and FVII deficiency, the association with AL amyloidosis has not been excluded or reported and is strongly suspected in the two cases of PCL here described in view of the presence of hepatosplenomegaly, ALP, and NT-proBNP elevation. The majority of patients with amyloidosis had multi-organ involvement. In addition to the liver and/or spleen, three patients had kidney involvement, two had cardiac amyloidosis (which was suspected in two further patients from our case records without a hystopathologically confirmed diagnosis), one had intestinal disease diagnosed on autopsy samples. The plasma cell disorders were associated with k light chain expression in three of the seven patients for whom the data are available, and with lambda light chain expression in three. In one case, no monoclonal band was detected on serum and urine electrophoresis. One case was associated with lambda light chain MM. This finding, despite the limits of the reduced sample size, argues against the association of the defect with a specific light chain type. In two cases, ≥5% circulating plasma cells were detected, consistent with PCL (primary and secondary, respectively). Age varied between 27 and 73 years. Six were male and two female. In one case, the age and sex were not reported.

## 4. Type and Severity of Clinical Manifestations and Outcome for Patients with Acquired Isolated FVII Deficiency Associated with Plasma Cell Disorders

Reported factor VII levels varied from 18% to 46% of normal; PT, measured in seconds, varied from 14 to 46. With the exception of two patients with a mild deficiency (FVII levels of 44% and 46% of normal levels, respectively) who showed no or mild bleeding tendency, the majority of cases (five patients) have been reported to be associated with severe bleeding, including delayed post-procedural bleeding (neck and retroperitoneal hematoma, and oral mucosal bleeding after a dental procedure) and spontaneous bleeding (nasal bleeding, pulmonary hemorrhage, and intracranial hemorrhage). Overall, there were three fatalities. Bleeding was the direct cause of death in two cases (intracranial hemorrhage in the context of interstitial pneumonia, upper GI bleeding; one patient died from infective complications after achieving hematological remission and correction of the clotting defect. Conversely, Girolami et al. reported that 8 of 29 patients with acquired FVII deficiency from any etiology had no bleeding tendency and that in the remaining patients, the entity of bleeding was variable and distributed between mild or moderate (8 cases) and severe (13 cases) and often out of proportion with the entity of the defect [10]. Furthermore, a study on 60 patients with AL amyloidosis-related isolated FX deficiency (≤50%) who underwent an invasive procedure reported that bleeding complications were relatively infrequent, particularly in patients undergoing nonvascular procedures, and that baseline factor X levels were not predictive of bleeding risk [18].

It is important to emphasize that in patients with plasma cell disorders, bleeding tendency may be enhanced compared to acquired isolated factor VII deficiency from other underlying conditions and due to inherited defects due to a number of concomitant factors: thrombocytopenia and platelet dysfunction (secondary to bone marrow insufficiency and/or chemotherapy), hyperfibrinolysis, hyperviscosity, vascular fragility, impaired vasoconstriction, renal failure, and thromboprophylaxis [19]. Moreover, infections that frequently complicate the course of disease in the immune-suppressed population may exacerbate coagulopathy and the risk of severe bleeding. In the case of pPCL presented in this paper, pancytopenia, including severe thrombocytopenia from massive plasma cell bone marrow infiltration, probably had an impact on the severity of bleeding; increasing serum monoclonal protein unresponsive to anti-MM therapy may also have contributed to bleeding tendency by causing hyperviscosity. Finally, the fatal bleeding event occurred during uncontrolled infection. Some of the above-mentioned factors may also account for the severity of hemorrhagic diathesis and the high mortality in patients with FVII deficiency after SCT, where bleeding was directly responsible for, or was a contributing factor to, death in a high proportion of patients [11].

Both in patients with an acquired defect from any etiology, including transplant patients, and particularly in plasma cell disorders, a decrease in FVII activity seemed to be associated with a poor prognosis [10,11]. In AL amyloidosis, FVII deficiency, without reference to whether it was isolated or combined, has been reported to be an independent predictor of death, similar to FX deficiency and prolonged PT [6]. The unfavorable outcome may be related not only to the risk of bleeding, but also to advanced disease and multi-organ involvement [5,6].

Although limited in size, the series reported here suggests that in patients affected by plasma cell dyscrasias, bleeding tendency might correlate with the activity of the factor tested as a percentage of normal activity levels. The correction of PT and factor VII deficiency after chemotherapy, surgery, or supportive measures corresponded, in fact, to a clinical response in terms of bleeding control. The previously mentioned review of acquired isolated FVII deficiency from any cause [10] considered the defect to be significant when lower than 40% of normal levels, and patients with levels higher than 40% were excluded from the analysis. Conversely, in the inherited disorder there is no direct correlation between the plasma levels of FVII and bleeding manifestations. Clinical phenotypes range from asymptomatic conditions—even in homozygous subjects—to severe, life-threatening bleeding [7,20].

## 5. Potential Mechanisms Underlying Acquired Isolated FVII Deficiency Associated with Plasma Cell Disorders

Proposed mechanisms for acquired FVII deficiency in plasma cell disorders are described in Table 2. FVII is disproportionately lower in chronic liver disease than other vitamin-K-dependent factors, probably due to its short half-life. However, none of the cases reported here showed signs of liver dysfunction at detection of factor VII deficiency and/or at onset of bleeding diathesis. Liver function tests were normal or near-normal in all patients, at least at the time of diagnosis of the clotting disorder, apart from an increase in ALP in patients with documented or suspected hepatic amyloidosis. PTT and the other vitamin-K-dependent (II, IX, and X) and -independent (V, VIII, XI, and XII) coagulation factors were also preserved when evaluated on repeated follow up. In the case of the patient with pPCL described here, in the face of signs of progressive liver dysfunction, likely secondary to progressive disease complicated by interstitial pneumonia, PT remained stable and PTT constantly remained within normal limits. The above observations suggest that the mechanism of factor VII deficiency in MM/AL amyloidosis is independent of liver function and the hepatic synthesis of clotting factors. Another clinical feature that points to accelerated clearance rather than synthetic dysfunction as the mechanism for FVII deficiency is the lack of response in FVII levels to adequate replacement therapy with FFP, full plasma exchange, and factor concentrate, as well as to parenteral vitamin K supplementation.

Notably, splenectomy after splenic rupture was reported to be beneficial in terms of the correction of factor deficiency and PT in two cases of amyloidosis [12], including one patient from our case records with complete normalization of PT documented as early as one week after surgery. Similarly, the resolution of FX deficiency has been described after splenectomy in patients with amyloidosis [21,22]. These observations suggest a similar etiology for these acquired clotting disorders, likely involving the binding of coagulation factors to splenic and hepatic amyloid deposits. However, a similar mechanism has not been proven to date for clotting factors other than FX.

Among other proposed mechanisms underlying an acquired FVII deficiency is an accelerated consumption where a massive, sudden availability of TF occurs, as in the case of increased leakage into surrounding tissue in patients who have undergone extensive chemotherapy, which is known to alter capillary permeability [11]. This may represent the mechanism underlying acquired FVII deficiency in patients undergoing SCT, as supported by the elevated incidence of veno-occlusive disease (VOD) of the liver in this clinical setting. The initial trigger for the development of VOD is thought to be the activation of liver sinusoidal endothelial cells and endothelial damage caused by toxic metabolites generated during conditioning regimens, with subsequent activation of the coagulation pathway [23]. Another example of increased catabolism or destruction may occur in cases of sepsis due to the proteases secreted by the leucocytes [24]. Among the above-mentioned mechanism of accelerated clearance, probably more than one acts in some patients with plasma cell disorders, particularly in the setting of chemotherapy/SCT or infections that frequently complicate the clinical course of the disease.

FVII deficiency in plasma cell dyscrasias does not appear to be related to the presence and amount of serum and urine monoclonal protein. This condition has been described in one case of light chain MM with no intact monoclonal immunoglobulin production [16] and in one patient with amyloidosis detected in tissue biopsy samples with no monoclonal band on serum and urine electrophoresis [15]. Elezovic et al. report treatment with plasmapheresis in an attempt to decrease paraprotein concentration in a patient with MM and amyloidosis and life-threatening bleeding, with very limited response in terms of rise in FVII and X levels. In line with the other reported cases, coagulation normalization was attained only after effective systemic chemotherapy, possibly as an effect of decreased new amyloid deposition in tissues. Notably, after chemotherapy discontinuation, clotting factor levels gradually decreased [17]. Consistently, the serum monoclonal protein increase in patient 1 from our case records did not translate into the further prolongation of PT.

PT mixing study with normal plasma, performed on one patients, corrected abnormal PT, indicating a simple clotting factor deficiency in the absence of an inhibitor. Notably, in the review by Girolami et al., the mixing study did not correct abnormal PT in 6 out of 25 cases that had been evaluated. An inhibitor was characterized in three of them. Underlying disorders associated with the presence of a FVII inhibitor included an autoimmune condition (aplastic anemia) in one case, and cancer, including one case of AML [10].

## 6. Therapeutic Considerations

As only few case reports exist in the literature, there is no consensus as to how patients with isolated acquired factor VII deficiency, and, more specifically, patients with underlying plasma cell disorders should be managed in terms of prophylactic and therapeutic measures. A few remarks can be made following the analysis of the series reported in this paper. Previously published cases, similarly to the personal cases described here, report inadequate response to FFP administration even at high doses. FFP may not be an effective therapy to correct factor VII deficiency due to the low concentration of FVII in plasma and its short half-life. In patients with amyloidosis, accelerated clearance by absorption of the factor by the tissue amyloid may also play a role [2].

In support of this assumption, the administration of factor concentrates in a patient with MM and AL amyloidosis with an acquired combined deficiency of FVII and FX resulted in a transient increase in the factor levels followed by a fall to baseline values within 30 min [17]. In a different clinical setting of accelerated FVII consumption as is the case of chemotherapy/SCT, the defect was uniformly refractory to parenteral vitamin K supplementation, massive FFP infusion and, in one case, to plasma exchange with FFP replacement. With regard to the inherited FVII deficiency, on the other hand, plasma has been successfully used to manage patients undergoing surgery, either by itself or in combination with FVII concentrate [25,26]. The daily transfusion of high doses of FFP may be complicated by fluid overload, especially in patients with cardiac amyloid involvement.

The use of recombinant activated factor VII (rFVIIa) has been reported to be of value in patients with plasma cell disorders and acquired FVII deficiency who are actively bleeding [14,16]. rFVIIa is indicated for use in the congenital deficiency of factor VII and acquired hemophilia, and patients with acquired inhibitors to hemophilia A and B replacement products [27]. Other varieties of uses, however, have been reported in diverse acquired factor VII and factor X deficiencies [28,29]. Interestingly, rFVIIa has been successfully used in the management of amyloid-associated factor X deficiency [30].

The use of prothrombin complex concentrate (PCC) has not been reported to date in patients with acquired isolated FVII deficiency from any cause [10], including patients with underlying plasma cell disorders. On the other hand, there are reports of the successful use of PCC for treatment and prophylaxis of bleeding, including the perioperative setting, in acquired FX deficiency associated with AL amyloidosis [31,32]. Among other therapeutic indications, PCC can be used for the treatment and prophylaxis of bleeding in congenital deficiency of any vitamin-K-dependent coagulation factors when purified specific products are unavailable. Moreover, four-factor PCC (i.e., PCC containing factors II, VII, IX, and X) has been demonstrated to decrease bleeding in the perioperative setting and in the emergent setting both in patients with coagulopathy, as evidenced by a prolonged PT, and those with a normal coagulation profile. Against this background, the use of four-factor PCC may be of value in acquired FVII deficiency, but clinical experience is needed.

The treatment or removal of the underlying cause, if this can be identified, is clearly important in all cases of acquired FVII deficiency [10]. More specifically, in the case of plasma cell dyscrasias, resolution of the bleeding disorder invariably requires treatment of the plasma cell clone by means of rapidly acting regimens, i.e., cyclophosphamide, bortezomib, and dexamethasone +/− daratumumab. The administration of immunomodulatory drugs (lenalidomide and thalidomide) in patients with MM may be challenging due to the contraindication to thromboprophylaxis in patients with a concomitant bleeding disorder. In the patient with pPCL from our case records, aspirin and thalidomide were immediately discontinued at the onset of bleeding. The choice of the therapeutic regimen should favor combinations with limited hematological toxicity, since thrombocytopenia may exacerbate the bleeding tendency. The failure to attain a hematologic response and, thus, to prevent new amyloid deposition in tissues translated into persistent and uncontrolled bleeding diathesis with fatal outcomes in two cases, including our personal case of pPCL [14]. On the other hand, in patients responsive to specific treatment, the time for FVII to recover to normal levels was reported to be 30–46 days, concomitant with therapeutic response [13,16] in two patients diagnosed with MM, and with no reported significant hepatosplenomegaly or documented amyloidosis. It is plausible that in patients with hepatosplenic amyloidosis, particularly in the presence of massive organomegaly, the clearance of factor-binding amyloid deposits and the subsequent correction of coagulation may take a prolonged time with negative impact on prognosis. In a patient with MM and amyloidosis with liver involvement, Elezovic et al. report a gradual rise in FVII and X with a resolution of bleeding diathesis after a total of nine monthly cycles of chemotherapy [17].

As previously mentioned, and in line with previous reports in patients with FX deficiency secondary to amyloidosis [21,22], splenectomy after splenic rupture has been reported to be beneficial in terms of resolution of the clotting disorder in two cases. In a patient from our case records, the complete correction of PT was documented as early as one week after splenectomy. Splenectomy might, therefore, represent an option in cases of intractable, life-threatening bleeding diathesis in the absence of a prompt response to specific plasma-cell-targeted treatment. Effective hemostasis might be provided by rFVIIa administration in the pre- and perioperative setting.

Finally, fibrinolytic inhibitors, i.e., tranexamic acid and fibrin glue, may be helpful in promoting and securing hemostasis.

In the future, a potential option to treat amyloid-related acquired factor FVII deficiency may be represented by monoclonal antibodies that bind to amyloid light chain fibrils, promoting the clearance from affected tissues [33]. A recent phase Ia/Ib clinical trial of CAEL-101 reported therapeutic responses in 63% of patients with cardiac, renal, hepatic, gastrointestinal, or soft tissue involvement. However, no specific data on acquired clotting factor deficiencies were reported. Treatment options for acquired FVII deficiency in plasma cell disorders are summarized in Table 3.

## 7. Conclusions and Final Considerations

This study emphasizes the importance of a careful evaluation of isolated prolonged PT and bleeding diathesis in patients with plasma cell disorders in view of the diagnostic, therapeutic and prognostic implications of acquired FVII deficiency. Acquired FVII deficiency should be considered a potential, although rare, complication adversely affecting the outcome of patients with MM and AL amyloidosis. The discovery of acquired FVII deficiency in a patient with MM or MGUS should prompt an evaluation for AL amyloidosis, particularly for amyloid hepatosplenic involvement, whenever not previously documented. Conversely, a comprehensive coagulation profile including clotting factor levels needs to be evaluated in a patient with suspected or documented hepatosplenic amyloidosis in order to adopt adequate prophylactic and therapeutic measures.

The treatment of the underlying plasma cell disorder is the mainstay of coagulopathy management and a rapidly effective therapy targeting the plasma cell clone should be instituted promptly in order to prevent severe bleeding complications. The introduction of anti-CD138 monoclonal antibodies in the treatment of MM and, more recently, amyloidosis [34,35], both in the first line and in the relapsed/refractory setting, represents a viable tool for achieving rapid plasma cell clearance and preventing further amyloid deposition in tissues which may be responsible for FVII sequestration. Monoclonal antibodies that bind to amyloid light chain fibrils promoting the clearance from affected tissues represent a potential option in a future perspective, but data need to be collected in this specific clinical setting [33]. Splenectomy has been reported to correct the clotting defect in patients with hepatosplenic amyloidosis. FFP replacement has been demonstrated to be ineffective in correcting acquired FVII deficiency and in preventing or treating bleeding complications. Published data, although on a limited number of cases, suggest that rVIIa may represent a useful supportive care measure, both in treating active bleeding and in providing effective hemostasis in the pre- and perioperative settings, including splenectomy. However, further clinical experience is needed to assess rVIIa efficacy and to optimize therapeutic management of this rare disorder.

## Figures and Tables

**Table 1:** Characteristics of patients with acquired factor VII deficiency and plasma cell disorders. The analysis also includes one patient with AL amyloidosis and isolated PT prolongation, whose coagulopathy resolved immediately after splenectomy, but who was not specifically tested for factor VII activity, and one patient with MM and AL amyloidosis with combined FVII and FX deficiency [17].

Author	Age, Gender	Diagnosis	AL Amyloidosis Organ Involvement	PT (Seconds, Ratio)	FVII (% of Normal)	Mixing with NP	Bleeding Tendency	Site of Bleeding	Underlying Disease Treatment	Supportive Measures	Comments
Present article, 2023	52, F	Primary PCL, IgA/ k	Suspected hepatosplenic and cardiac (massive hepatosplenomegaly, increased ALP and NT-proBNP). Liver biopsy NA	NR, 1.6	22	Corrected	Severe	Epistaxis, cutaneous, intracranial	D-VTd	FFP, tranexamic acid	Unresponsive to FFP, IV vitamin K, anti-MM therapy. Fatal (massive intracranial hemorrhage, CMV pneumonia)
Present article, 2023	54, M	Secondary PCL, IgG/λ	Suspected hepatosplenic and cardiac (splenomegaly, increased ALP, NT-proBNP). Abdominal fat biopsy negative. Liver biopsy NA	NR, 1.85	44	Corrected	Mild	Cutaneous (easy bruising)	Dexamethasone and Cyclophosphamide; Talquetamab (4th and 5th line of therapy, respectively)	FFP, tranexamic acid	Unresponsive to FFP, IV vitamin K. Corrected 30 days after start of anti-MM therapy; PT prolonged with sepsis
Present article, 2023	44, M	AL amyloidosis, λ	Splenic, renal	NR, 1.4	NA	NA	No bleeding symptoms	-	Splenectomy after splenic rupture. D-CyBorD	None	FVII levels NA. PT corrected 7 days after surgery
Elezovic et al., 1989 [17]	50, M	MM,IgG/k + AL amyloidosis, k	Hepatic, renal, intestinal.Dx on BM biopsy and autopsy	Combined FVII and FX deficiency PTT 101 s, PT 46 s	FVII 0.25 U/mL (nv 0.5–1.5) FX 0.01 U/mL (nv 0.5–1.5)	Corrected (PT and PTT mixing test)	Severe	Epistaxis, soft tissue, retroperitoneal	VMCP ABP	FFP, factor concentrates	Unresponsive to FFP, factor concentrates, IV vitamin K. Recovery after 9 months of anti-MM therapy. Death due to infective complications
Uematsu et al., 1997 [12]	27, F	AL amyloidosis	Splenic. Dx on splenectomy	22 s, NR	18	Corrected	Severe	NR	Splenectomy after splenic rupture	NR	Improved after surgery
Hu et al., 2014 [13]	6 cases of MM presenting with coagulopathy, including 1 case of isolated FVII deficiency and 5 with complex factor deficiencies	MM	NA	NR	29	NR	NR	NR	Anti-MM treatment, unspecified	NR	Median time to coagulation recovery: 46 days
Nguyen et al., 2018 [14]	52, M	AL amyloidosis, k	Hepatosplenic (massive hepatosplenomegaly) and cardiac. Dx on liver biopsy	13.9 s, 1.5	29	Corrected	Severe	Post-procedural (neck hematoma, oral mucosal, retroperitoneal); GI	CyBorD	FFP, DDAVP, VWF/FVIII concentrate, aminocaproic acid, rVIIa	Unresponsive to treatment.Fatal (uncontrolled upper GI bleeding)
Dursun et al., 2018 [15]	58, M	AL amyloidosis. Serum/urine monoclonal band not detected	Hepatosplenic (massive hepatosplenomegaly), cardiac, renal. Dx on renal and BM biopsy	17.9 s, 1.48	46	Corrected	No bleeding symptoms (spontaneous or post-procedural)	-	CyBorD	-	Unresponsive to IV vitamin K. Recovery after 3 months of anti-amyloidosis therapy
Zaidi et al., 2019 [16]	73, M	MM, λ light chain	NA	15 s, NR	34	NR	Severe	Pulmonary	Bortezomib-based, unspecified	rFVIIa	Recovery of FVII to 70% after 1 month of anti-MM therapy

Abbreviations: ABP = Adriamycin, Belustin, and Prednisone; BM = bone marrow; CMV = Cytomegalovirus; CyBorD = Cyclophosphamide, Bortezomib, and Dexamethasone; DDAVP = desmopressin; Dx = diagnosis; FVII = factor VII; D-CyBorD = Daratumumab, Cyclophosphamide, Bortezomib, and Dexamethasone; D-VTd = Daratumumab, Bortezomib Thalidomide, and Dexametahasone; FFP = fresh frozen plasma; GI = gastrointestinal; MM = multiple myeloma; PCL = plasma-cell leukemia; PT = prothrombin time; rFVIIa = recombinant activated factor VII; NA = not assessed; NP = normal plasma; NR = not reported; VMCP =Vincristine, Melphalan, Cyclophosphamide, and Prednisone.

**Table 2:** Proposed mechanisms for acquired FVII deficiency in plasma cell disorders.

	Comments
Decreased hepatic synthesis of FVII	
	The mechanism of isolated FVII deficiency in plasma cell disorders is largely independent of hepatic synthesis (PTT and the other vitamin-K-dependent and independent coagulation factors are preserved; FFP/plasma exchange, factor concentrate, parenteral vitamin K are ineffective in correcting coagulation) Hepatic failure secondary to progressive disease (AL amyloidosis, plasma cell infiltration of the liver) may exacerbate the clotting defect through decreased synthesis. FVII is disproportionately lower in chronic liver disease than other vitamin-K-dependent factors due its short half-life
**Accelerated clearance/catabolism of FVII**	
Binding of FVII to splenic and hepatic amyloid deposits	This mechanism has not been provedn to date for clotting factors other than FX It may be postulated based on the prompt correction of PT in response to splenectomy in patients with splenic amyloidosis, similar to FX deficiency, and based on the ineffectiveness of FFP/plasma exchange and factor concentrate
Increased tissue leakage of TF due to altered capillary permeability after chemotherapy/SCT	Chemotherapy/SCT may contribute to decreasing FVII levels through increased consumption
Increased destruction of FVII due to the proteases secreted by the leucocytes during sepsis	Infection may contribute to decreasing FVII levels through increased destruction
FVII inhibitor	The presence of an inhibitor has never been demonstrated to date in patients with plasma cell disorders and acquired isolated FVII deficiency

Abbreviations: FFP = fresh frozen plasma; FVII = factor VII; FX = factor X; PTT = partial thromboplastin time; SCT = stem cell transplant; TF = tissue factor.

**Table 3:** Therapeutic options for acquired FVII deficiency in plasma cell disorders.

Treatment	Pros	Cons
Treatment of the underlying plasma cell disorder [12,13,14,15,16,17]	Potentially resolutive, should be instituted promptly in all cases	Prolonged time is generally required to correct coagulation, particularly in the presence of hepatosplenic amyloidosis Hematological toxicity, i.e., thrombocytopenia secondary to anti-MM agents, may exacerbate bleeding diathesis Regimes that are rapidly acting in terms of FLC clearance (i.e., Cyclophosphamide, Bortezomib, and Dexamethasone +/- Daratumumab) and with limited hematological toxicity should therefore be preferred IMIDs may be challenging to use due to the contraindication to thromboprophylaxis in patients with coagulopathy CT may increase FVII consumption from altered capillary permeability and tissue leakage of TF CT may increase the risk of infection and FVII destruction due to the proteases secreted by the leucocytes
Splenectomy [12]	Potentially resolutive in patients with splenic amyloidosis Rapidly acting in terms of correction of coagulation	Invasive procedure associated with potential bleeding risk. rFVIIa, PCC, and/or other supportive measures are indicated in the pre- and perioperative setting
Monoclonal antibodies promoting the clearance of amyloid light chain fibrils from tissues [33]	Potentially resolutive in patients with amyloidosis Potentially rapidly acting	Investigational. No specific data on acquired clotting factor deficiencies have been reported to date
rFVIIa [14,16]	Supportive measure Effective as hemostatic agent in treating active bleeding and in the pre- and perioperative setting	Risk of thromboembolic events Off-label use
Prothrombin complex concentrate (four-factor)	Supportive measure Potentially effective as hemostatic agent in treating active bleeding and in the pre- and perioperative setting	No data on patients with FVII deficiency associated with plasma cell disorders have been reported to date Risk of thromboembolic events Off-label use
FFP/plasma exchange [14,17]	Supportive measure	Limited efficacy as a result of low concentration of FVII in plasma, short half-life, and accelerated FVII clearance in amyloidosis Risk of fluid overload
Factor VII concentrate [17]	Supportive measure	Limited efficacy as a result of short half-life and accelerated FVII clearance in amyloidosis
Tranexamic acid	Supportive measure	May contribute to hemostasis both in the setting of treatment of active bleeding and prophylaxis
Parenteral vitamin K [15,17]		Ineffective. The mechanism of acquired FVII deficiency in plasma cell disorders is largely independent of hepatic synthesis

Abbreviations: CT = chemotherapy; FVII = factor VII; FLC = free light chains; FFP = fresh frozen plasma; IMIDs = immunomodulatory drugs; MM = multiple myeloma; PCC = prothrombin complex concentrate; rFVIIa = recombinant activated factor VII; TF = tissue factor.

## Data Availability

The data presented in this study are available on request from the corresponding author.
